# Machine-Guided Discovery of Acrylate Photopolymer Compositions

**DOI:** 10.1021/acsami.4c00759

**Published:** 2024-03-27

**Authors:** Ayush Jain, Connor D. Armstrong, V. Roshan Joseph, Rampi Ramprasad, H. Jerry Qi

**Affiliations:** † School of Material Science and Engineering, Georgia Institute of Technology, Atlanta, Georgia 30332, United States; ‡ College of Computing, Georgia Institute of Technology, Atlanta, Georgia 30332, United States; § School of Mechanical Engineering, Georgia Institute of Technology, Atlanta, Georgia 30332, United States; ∥ Renewable Bioproducts Institute, Georgia Institute of Technology, Atlanta, Georgia 30332, United States; ⊥ H. Milton Stewart School of Industrial and Systems Engineering, Georgia Institute of Technology, Atlanta, Georgia 30332, United States

**Keywords:** additive manufacturing, 3D printing, photopolymers, material discovery, active learning

## Abstract

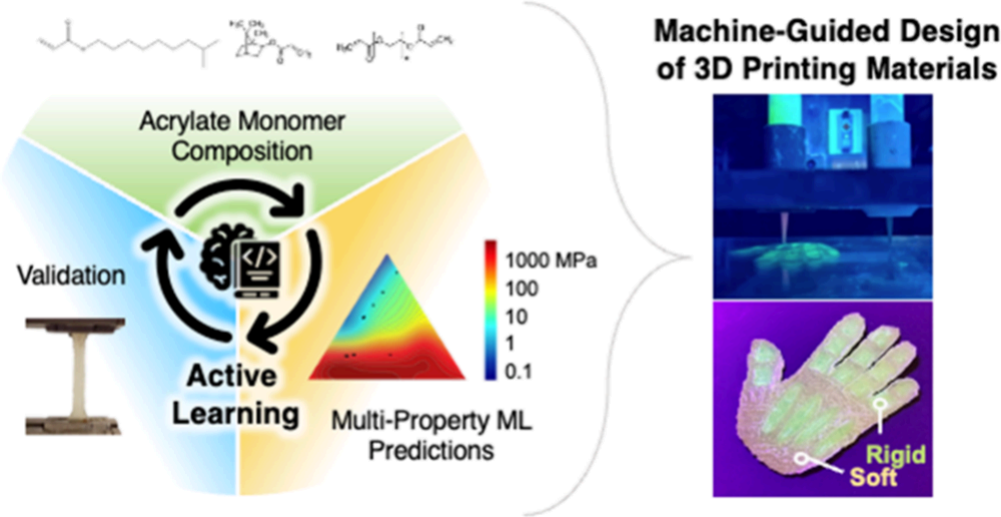

Additive manufacturing (AM) can be advanced by the diverse characteristics offered by thermoplastic and thermoset polymers and the further benefits of copolymerization. However, the availability of suitable polymeric materials for AM is limited and may not always be ideal for specific applications. Additionally, the extensive number of potential monomers and their combinations make experimental determination of resin compositions extremely time-consuming and costly. To overcome these challenges, we develop an active learning (AL) approach to effectively choose compositions in a ternary monomer space ranging from rigid to elastomeric. Our AL algorithm dynamically suggests monomer composition ratios for the subsequent round of testing, allowing us to efficiently build a robust machine learning (ML) model capable of predicting polymer properties, including Young’s modulus, peak stress, ultimate strain, and Shore A hardness based on composition while minimizing the number of experiments. As a demonstration of the effectiveness of our approach, we use the ML model to drive material selection for a specific property, namely, Young’s modulus. The results indicate that the ML model can be used to select material compositions within at least 10% of a targeted value of Young’s modulus. We then use the materials designed by the ML model to 3D print a multimaterial “hand” with soft “skin” and rigid “bones”. This work presents a promising tool for enabling informed AM material selection tailored to user specifications and accelerating material discovery using a limited monomer space.

## 1 Introduction

Thermoplastic and thermoset polymers offer a wide range of material characteristics that are useful for additive manufacturing (AM).^1−3^ For instance, copolymerization with multiple monomers leads to millions of possible materials. Currently, polymeric material selection for AM is predominantly limited to those with well-documented material characteristics, which may not be entirely appropriate for desired applications.^4,5^ Moreover, due to the tremendous number of possible combinations of monomers, lengthy and costly experimental characterization has become impractical for the discovery of new AM material chemistries.^6,7^ Machine learning (ML)-based informatics approaches offer a promising avenue for targeted design via accelerated data-driven polymer property prediction and guiding successive rounds of experimentation.^8−10^ However, a major bottleneck for the wide adoption of ML in AM -as well as in many subfields of material research - is the need for sufficiently large and, more importantly, diverse, datasets.^11,12^

A solution to this problem that has made major inroads in recent years is active learning (AL), which is a process to build an ML model (and dataset) iteratively from a small starting set of training points using the principles of Bayesian optimization (BO).^13−16^ This creates a path to obtain informed recommendations for successive experiments, minimizing the number of experiments required (to meet a target material or performance metric) and thus expediting the development of new materials. AL has been used to accelerate material design problems in polymers, alloys, and ceramics.^17−21^ Within the AM space, ML has been used to enhance *in situ* process monitoring to improve print quality through optimizing printing parameters, resin composition, and component attributes.^19,22−24^ AL has been used to accomplish such tasks with automated decision making.^22,23,25^ A small subset of recent literature deals with the modeling of acrylates for several properties, albeit in a limited capacity. Notable contributions include the modeling of glass transition temperatures for copolymers, tested on an expansive acrylate dataset, and the application of physics-constrained BO to enhance tensile strength and toughness of thermoplastic polymers.^26,27^ The expanding literature highlights the potential for further investigation into optimizing multiple acrylate properties in data-scarce situations using novel data recommendation approaches and ML algorithms. Additionally, the variability of experimental data is often disregarded in the AL cycles of previous studies, especially within the data recommendation method, which selects the next set of experiments. Choosing experiments without accounting for the variability across trials can be risky.^28^ Novel data recommendation methods, such as Noisy Expected Hypervolume Improvement (NEHVI), have been proposed to account for this variability during sample selection in BO and tested theoretically in other domains.^29,30^ NEHVI has not been explored in material design but holds promise for AL to search material design spaces with high variability.

**Figure 1:**
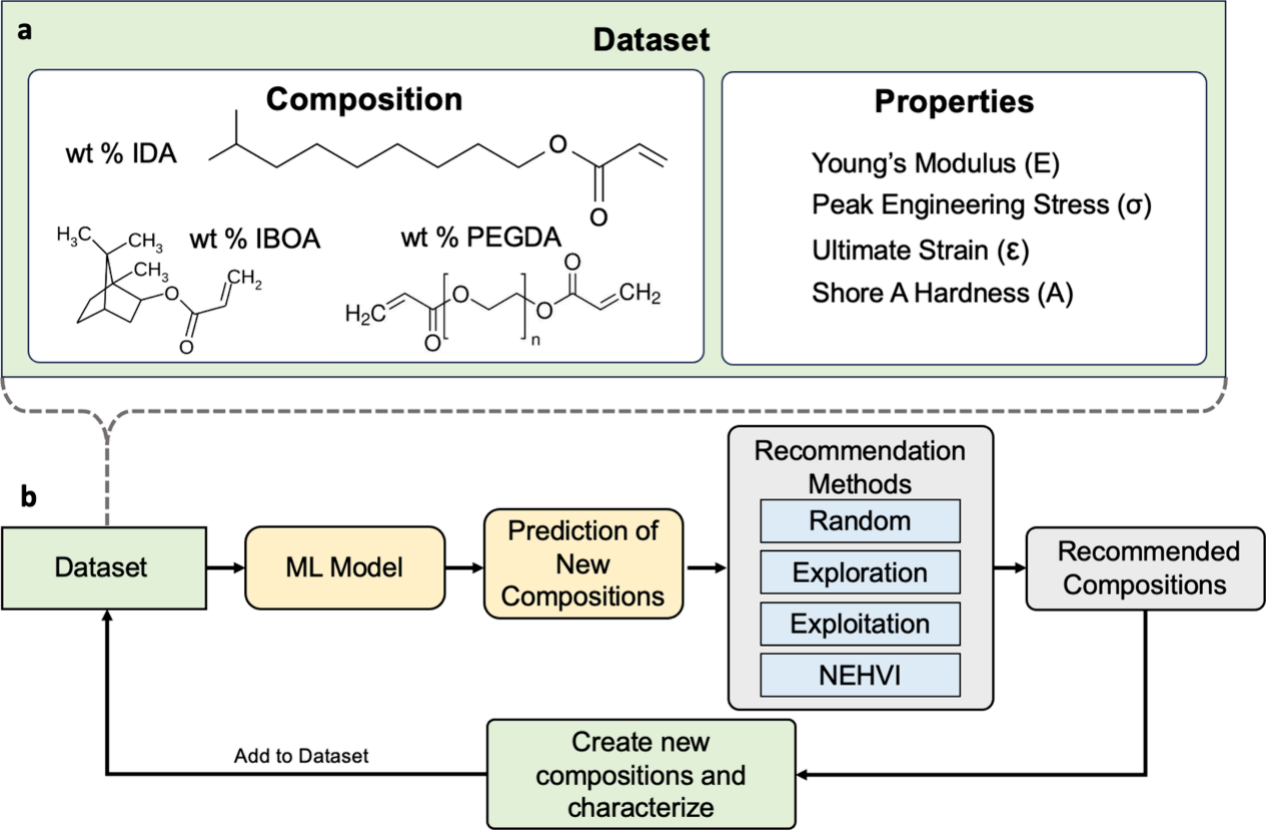
Input materials and active learning loop. (a) Structure of investigated monomers: isodecyl acrylate (IDA), isoborynol acrylate (IBOA), and poly(ethylene glycol) diacrylate (PEGDA). Properties characterized were Young’s modulus, peak engineering stress, ultimate strain, and Shore A hardness for every composition ratio. (b) Flowchart outlining the active learning process.

In this work, we introduce an AL approach to efficiently recommend multimonomer resins to provide improvement of desired material properties that surpasses improvement from uninformed experiments. Our method seeks to build an accurate predictive model while minimizing the number of experiments required to explore a composition space for targeted material design. To test our approach, we select a design space consisting of three free radical polymerizing monomers (Figure 1a) ranging from rigid to elastomeric (Section 2.1). The properties of interest are Young’s Modulus (*E*), peak engineering stress (σ), ultimate strain (ε), and Shore A hardness. Our AL approach detailed in Figure 1b begins with an initial training dataset, which are used to train a Gaussian Process Regression (GPR) model.^31^ The GPR model is used to predict the specified material characteristics of all possible monomer compositions in the design space (Section 2.2). Next, we used NEHVI to select several high-quality compositions to synthesize and characterize (Section 2.3). The selected compositions are then added to the dataset, and the cycle is repeated for five iterations, followed by a final uncertainty-based exploration selection to preclude regional inconsistencies. Moreover, we demonstrate the ability to accurately predict and subsequently recommend monomer compositions using fine-tuned hierarchical ML models and an exploitation recommendation (Section 2.4). This method recommends compositions with desired numerical values of Young’s moduli within 10% accuracy. Finally, we demonstrate a potential application of this technique to additive manufacturing through the multimaterial fabrication of a hand with “skin” and “bones”.

## 2 Experimental Methods and Materials

### 2.1 Chemical Space

In this work, we use three acrylate monomers to test our methodology: isodecyl acrylate (IDA), isoborynol acrylate (IBOA), and poly(ethylene glycol) diacrylate (PEGDA) (Sigma-Aldrich, St. Louis, MO, USA). These monomers were selected for their diversity of properties. Specifically, IDA exhibits elastomeric behavior due to its long 12 carbon chains, IBOA is highly rigid due to a high-molecular-weight and bulky functional group, and PEGDA acts as a cross-linker. Additionally, these polymers have a high compatibility when forming cross-linked heteropolymers. These monomers were mixed at specific weight composition ratios with 1 wt % photo initiator Irgacure 819 (bis(2,4,6-trimethylbenzoyl)-phenylphosphineoxide) (Sigma-Aldrich). The structures can be found in Figure 1a. The molecular weights for IBOA, IDA, and PEGDA are 208.3, 212.33, and 250 g/mol, respectively. Compositions with PEGDA greater than 60 wt % were not considered in this study because excessive cross-linking diminishes the contributions of other monomers, resulting in undesirable homogeneity in the characterization space.

### 2.2 ML Model

The ML model used in AL cycles is Gaussian Process Regression (GPR) due to its low training cost, ability to provide inference with fewer data points, and Bayesian interpretation.^17,31,32^ Inference as a distribution is a crucial part of BO,^16^ which will be elaborated in Section 2.3. We build models for four properties: *E*, ε, σ, and Shore A hardness; the model inputs are the monomer composition ratios. In this Bayesian approach, each property is modeled as a function of the monomer composition ratio. This underlying function is learned by the GPR model when trained on the experimentally obtained property values of the selected monomer compositions and the errors of the experiments. For new, unseen composition ratios, the GPR gives the posterior distribution of the underlying function. The mean of the posterior distribution can be used as the predicted value for the property, while the variance quantifies its uncertainty. A higher variance of the posterior distribution indicates a higher uncertainty for the prediction.

Each iteration of the AL cycle starts with training the GPR models on measured values of the properties of the considered compositions. Given a vector that contains the percentage composition of IDA, IBOA, and PEGDA, the models predict property values for unknown samples. Given these predictions, a recommendation method is used to find promising compositions, based on which samples are synthesized and characterized (see Section 2.3 for details). The property values for these samples and their uncertainties are added to the dataset for the ML model training in the next AL iteration cycle.

For quantifying the errors of the ML models, we use Root Mean Squared Error (RMSE) for linear scaled properties (hardness), whereas we use the Order of Magnitude Error (OME) for log-scaled properties such as *E*, σ, and ε (discussed in Section 2.4). The OME is calculated as the Mean Absolute Error of the log-scaled values.

### 2.3 Composition Recommendation Methods

Data quality is fundamental to ML; therefore, it is vital to use the right recommendation method to collect the highest quality data points for the best results from AL. We consider four classes of recommendation methods: (i) random sample, (ii) exploration, (iii) exploitation, and (iv) expected improvement (EI).^18,32^ For the random sample method, the model randomly selects the sample points for each iteration. The exploration method uses the variance of the posterior distribution of GPR to identify compositions for which the model is most uncertain, ignoring the mean of the model’s posterior.^18^ Alternatively, the exploitation method relies heavily on the model being well informed. The use of exploitation also requires desired conditions for compositions to achieve such as a target property value or a need to maximize or minimize a property. The compositions that the model predicts closest to this goal are selected. Exploitation ignores the variance component of the model posterior. Finally, the EI method balances the exploration and exploitation methods and locates samples with high uncertainty, which have a higher likelihood of achieving usable material characteristic goals. This is accomplished by finding compositions with high expectation for property value improvement relative to the best performing composition in the dataset of the present AL cycle. The EI criterion for maximizing a property, given a predicted value with mean (*y*_pred_(*x*)) and variance (σ^2^(*x*)) for a composition *x*, is defined as the expectation of improvement (*E*[*I*(*x*)]) of the predicted value from the best property value seen in the dataset (*y*_best_),
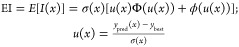
1where Φ(·) is the cumulative distribution function of the standard normal distribution and ϕ(·) is the standard normal density function.^33^

Our goal requires a recommendation based on four property objectives; however, the conventional EI eq (eq 1) is only suitable for one objective. As a result, past research has expanded EI approaches to many objectives using a recommendation method known as expected hypervolume improvement (EHVI).^30^ The hypervolume represents the overall quality of a sample and is calculated by the multiplication of each objective value scaled between 0 and 1. Therefore, a composition, which has a greater hypervolume than data points in the current AL cycle, has a higher likelihood of increasing the hypervolume. However, in our case, experimental error in the dataset affects the true value of hypervolume criteria. To account for this, we can treat the experimental error as a random variable with a normal distribution. Recall that the GPR provides the posterior distribution of a property, which is normally distributed. Integrating EHVI over these normal distributions yields a quantity defined as noisy expected hypervolume improvement (NEHVI).^29^ However, NEHVI of a composition *x* is not analytically solvable, so a Monte Carlo approximation (α̂_NEHVI_(***x***)) is used and presented in eq 2:^29^
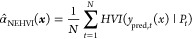
2where for *t* =1···*N,* a set of possible characteristic values (*N* = 100) is sampled from predicted normal distributions (*y*_pred,*t*_(*x*)) and experimental normal distributions. *P_t_* is the group of measured dataset values in the current AL cycle, in which no objective can be improved without sacrificing another (formally known as the pareto front). Intuitively, the composition ratios in *P_t_* maximize the hypervolume of the dataset and are analogous to *y*_best_ in eq 1. α̂_NEHVI_(***x***) is calculated by averaging over the hypervolume improvements (HVI) of every set of *y*_pred,*t*_(*x*) against its given *P_t_*.^29^ The NEHVI recommendation method represents an intuitive approach to choose compositions for our context by accounting for multiple noisy properties in experimental data.^29^

### 2.4 Approach for Targeted Property Predictions

For specific use cases, we might want to achieve a specific numerical value for a property that we call a “target value” (*y*_target_). To account for experimental error, we set a tolerance (ε_tol_) range that extends above and below the *y*_target_. For example, the *y*_target_ and ε_tol_ used in Section 3.2 are *E* = 1100 ± 100 MPa.

A limitation of the current implementation of the NEHVI recommendation method is that it is focused on maximizing the hypervolume of objectives but does not explicitly account for targeted objectives. To use the NEHVI recommendation when one or more objectives have targeted values, we can transform *y*_pred_ to center around the *y*_target_. In other words, we want to minimize
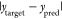


To use this as an objective for hypervolume, we apply a Gaussian transformation to obtain the targeted objective function (*f*_target_(*y*_pred_)), which we want to maximize,
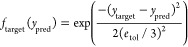


Intuitively, this function is shaped like a bell curve, where *f*_target_(*y*_*target*_) = 1. As *y*_pred_ moves away from *y*_target_, *f*_target_(*y*_pred_) gradually decreases to 0. *e*_tol_ determines how quickly or slowly this decrease occurs, controlling the “width” of the bell curve. Smaller *e*_tol_ values lead to a sharper decline, while larger values create a more gradual descent, as predictions deviate from the target. Tuning *f*_target_ in this way allows one or more targeted objectives to be used in hypervolume and NEHVI calculations.

We also change our process of predicting *y*_pred_ of characteristics with targeted objectives. As our composition space consists of rigid and elastomeric materials, the material characteristics of interest often span several orders of magnitude. This results in a data distribution that is skewed toward lower orders of magnitude. Using a linear scale for this type of data may impact the performance of the GPR model, which can be rectified by transforming the dataset.^34^ For this study, material characteristic values for *E*, σ, and ε are logarithmically scaled, improving GPR accuracy on the overall dataset. However, to target a particular numerical value of a material characteristic, GPR predictions need to be accurate in ranges close to this target value, which is difficult to achieve using logarithmically scaled values. Thus, we use a two-stage hierarchical approach (detailed in Figure S1) to make fine-tuned predictions on compositions that are predicted to have a property near the target value. The first stage uses a GPR model trained on the complete logarithmically scaled property set to make predictions for all possible compositions. The compositions that have a predicted property value near the target value are selected from the initial composition set. The second stage uses a GPR model to predict the property values of the selected compositions. This second GPR is trained on a linear scale on only compositions that are close to the target value. This method of obtaining *y*_pred_ yields more accurate predictions near *y*_target_. In Section 3.2, we demonstrate the use of this strategy.

### 2.5 Code Implementation

This work is implemented by using Python 3.8.7. The *BoTorch* library, a BO framework built on *Pytorch*, includes the NEHVI function implementation used in this study. We employ the *GPyTorch* library, a Gaussian Process framework also built on *Pytorch*, for the GPR model training. Because of the large computational cost of NEHVI, a GPU with CUDA 11.0 is used to execute the active learning loop.

### 2.6 Mechanical Characterization

Tensile samples were prepared by incrementally adding liquid resin into silicone molds and subjected to 5 s of UV irradiation with light intensity of 50 mW/cm^2^. This incremental procedure was repeated four times to ensure the sample was fully cured throughout. We performed tensile tests (*n* = 8) using a universal mechanical testing machine (Criterion, MTS, Eden Prairie, MN, USA) for each composition to characterize mechanical properties of the resins: Young’s modulus (E), peak engineering stresses (σ), ultimate strain (ε), and Shore A hardness. In this pilot study, we elected to perform experiments using solely cast dogbone samples to control for potential property variability introduced during the printing process including layer inhomogeneity or voids caused by toolpath motion.

### 2.7 Multimaterial 3D Printing

Separate 3D models representing the “skin” and “bone” of the multimaterial hand were created using SolidWorks (Dassault Systèmes SE, Vélizy-Villacoublay, France) computer-aided design (CAD) software. The two models corresponding to different materials were imported into the computer-aided manufacturing (CAM) software, Repetier (Hot-World GmbH & Co. KG, Willich in North Rhine-Westphalia, Germany) where they were positioned relative to one another in the desired print configuration. Each model was assigned a separate extruder, and therefore material, and were sliced into discrete layers. The printing parameters used are as follows: an extrusion pressure of 70–75 kPa, a layer thickness of 0.4 mm, a layer UV cure time of five seconds, and an extrusion nozzle deposition speed of 10 mm/s.

Selected liquid resins were mixed with fumed silica (Sigma-Aldrich) as a rheological modifier to facilitate shear-thinning behavior necessary for direct ink write 3D printing. Once prepared, the inks were loaded into syringes and mounted to a custom 3D printer, and the material deposited onto a glass substrate.^35^

## 3 Results and Discussion

Our AL iterations involved three major phases: dataset building, validation, and application. The first phase executed AL iterations using the parallel NEHVI recommendation method to build a dataset. In the second phase, we collected additional data to validate our approach and compared it to an uninformed recommendation method. The third phase used a targeted approach to select samples with a specific *E* for a specific use case. Figure S2 outlines every round of data collection and their purposes. We discuss the results from each phase in the proceeding sections.

### 3.1 AL Iterations for Dataset Building and Validation

**Figure 2:**
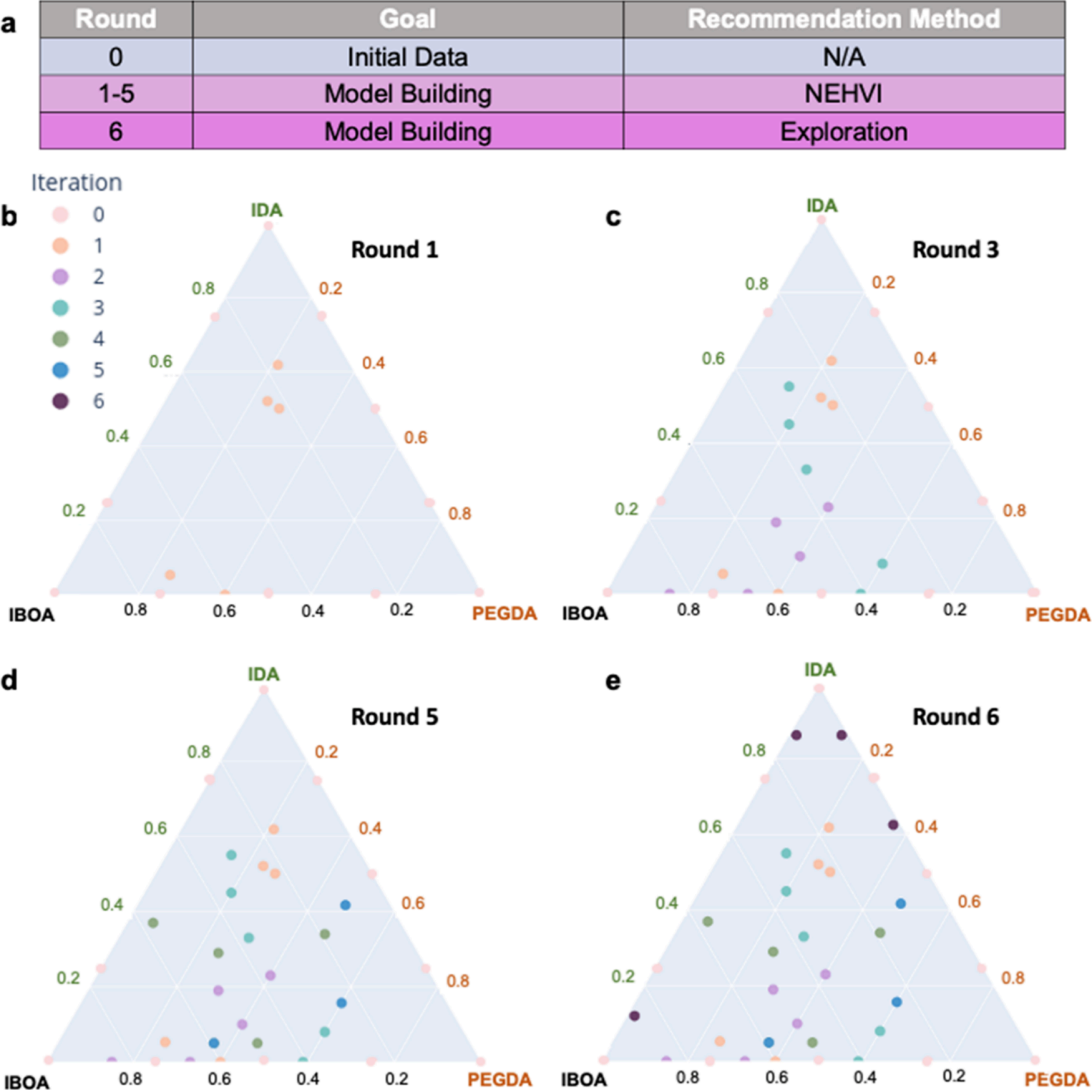
Building the dataset via AL using our informed recommendation approach. (a) Procedure for model building over six iterations. Experimental data space evolution was observed at (b) round 1, (c) round 3, (d) round 5, and (e) round 6.

**Figure 3:**
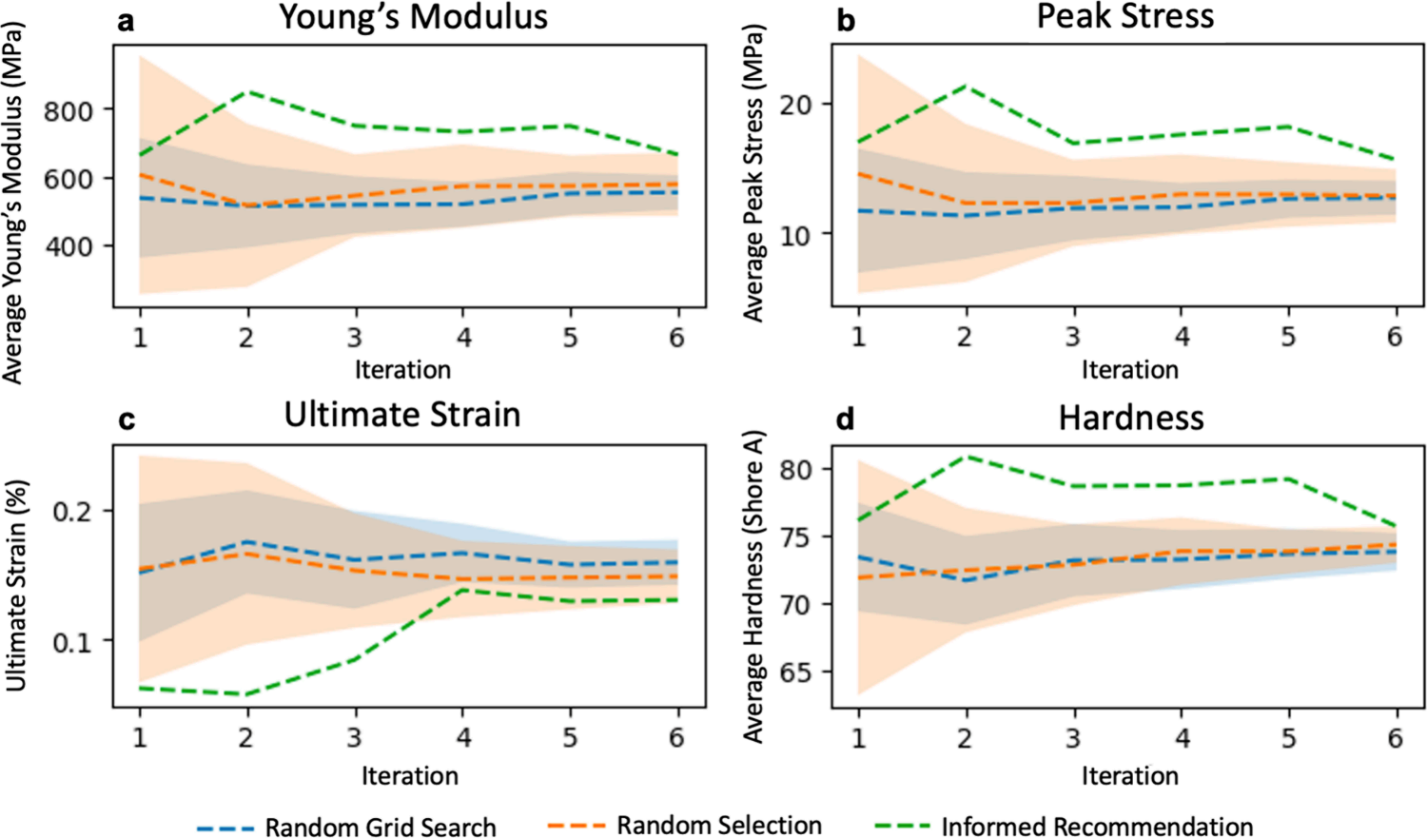
Effectiveness of the informed recommendation (Figure 2) is evaluated by comparing the average measured property values of the compositions added to the dataset with those added by random uninformed methods during the virtual experiments. The results show that the informed recommendation builds a dataset, which has (a) a maximized *E*, (b) a maximized σ, (c) a minimized ε, (d) and a maximized hardness. Shaded areas convey the standard deviations of values for 20 virtual experiments.

We started with an initial set of 11 discrete monomer compositions. The remaining dataset was built over six AL iterations (Figure 2a), with five monomer compositions recommended at each iteration. All five multimonomer compositions were created and characterized; however, some recommended compositions were unable to cure to the degree required for mechanical characterization. Therefore, these composition ratios could not be added to the dataset. The first five iterations used the parallel NEHVI recommendation (eq 2, Figure 2b–d), followed by a final iteration using an exploration recommendation method using only uncertainty values (Figure 2e). This final iteration was conducted to put emphasis on any regimes of compositions that remain in high uncertainty. We measured the quality of a collected dataset using two criteria: accuracy of the trained ML model (measured with RMSE and OME) (Figure S4) and average value of properties added to the dataset (Figure 3). The GPR model error exhibited an overall linear decrease across all four measured material characteristics (*R*^2^ = 0.81, 0.99, 0.83, and 0.55 for *E*, σ, ε, and hardness, respectively). Indeed, model error decreased by 44, 50, 46, and 20% from iteration 1 to 6 for *E*, σ, ε, and hardness, respectively.

After iteratively obtaining a dataset through our informed model building approach, we obtained a total of 30 recommended composition ratios, of which we were able to create and characterize 27, thus having a dataset of 39 data points, in addition to five randomly picked data points for validation. This prompts us to compare our approach to an uninformed technique, such as a grid search or a manual handpicking approach. To establish the validity of our approach against a method we term “random grid search” (detailed and described in Figure S3), we conducted a set of virtual experiments. In these experiments, we initiated a new GPR model and employed AL with the random grid search recommendation method to collect five compositions per iteration over six iterations. Since this was a purely virtual demonstration, we did not physically recharacterize new compositions. Instead, we exposed the GPR model to the previously characterized compositions within our candidate set of compositions and their properties. This set included the 45 compositions acquired during model building, alongside 11 hand-picked selected for characterization (Figure S2). The hand-picked points served to mitigate the bias of solely collecting from our existing well-informed data points proposed during the model building approach. We ran 20 trials of the virtual experiments for random grid search and a naïve random selection of compositions. Figure 3 presents a comparison between the mean and standard deviations of accuracy on validation points for both virtual experiments and our model building approach.

As a demonstration, our NEHVI approach was used to recommend compositions with rigid properties for structural applications. Thus, we chose NEHVI targets that are high *E*, high Shore A hardness, high σ, and low ε. In Figure 3, compositions recommended by NEHVI on average had 38.4, 47.7, and 6.9% higher *E*, σ, and hardness, respectively, and a 37.7% lower ε than the compositions recommended by random grid search. This provides an example of how the NEHVI recommendation approach can be used to find compositions with the desired characteristics in a design space.

Figure S4 compares the mean and standard deviations of accuracy on validation points between virtual experiments of random grid search and our model building approach. After six iterations of every virtual experiment, the average accuracy of the random grid selection method appears to be on par with or slightly better than that of our approach upon initial inspection. However, this is based on a series of 20 trials, revealing a notable variance in outcomes. In practice, we would have only one set of experiments to fully explore a space. Under this constraint, achieving a usable level of model accuracy across all four properties and a dataset with desired material characteristics using an uninformed approach is improbable compared to our informed approach.

**Figure 4:**
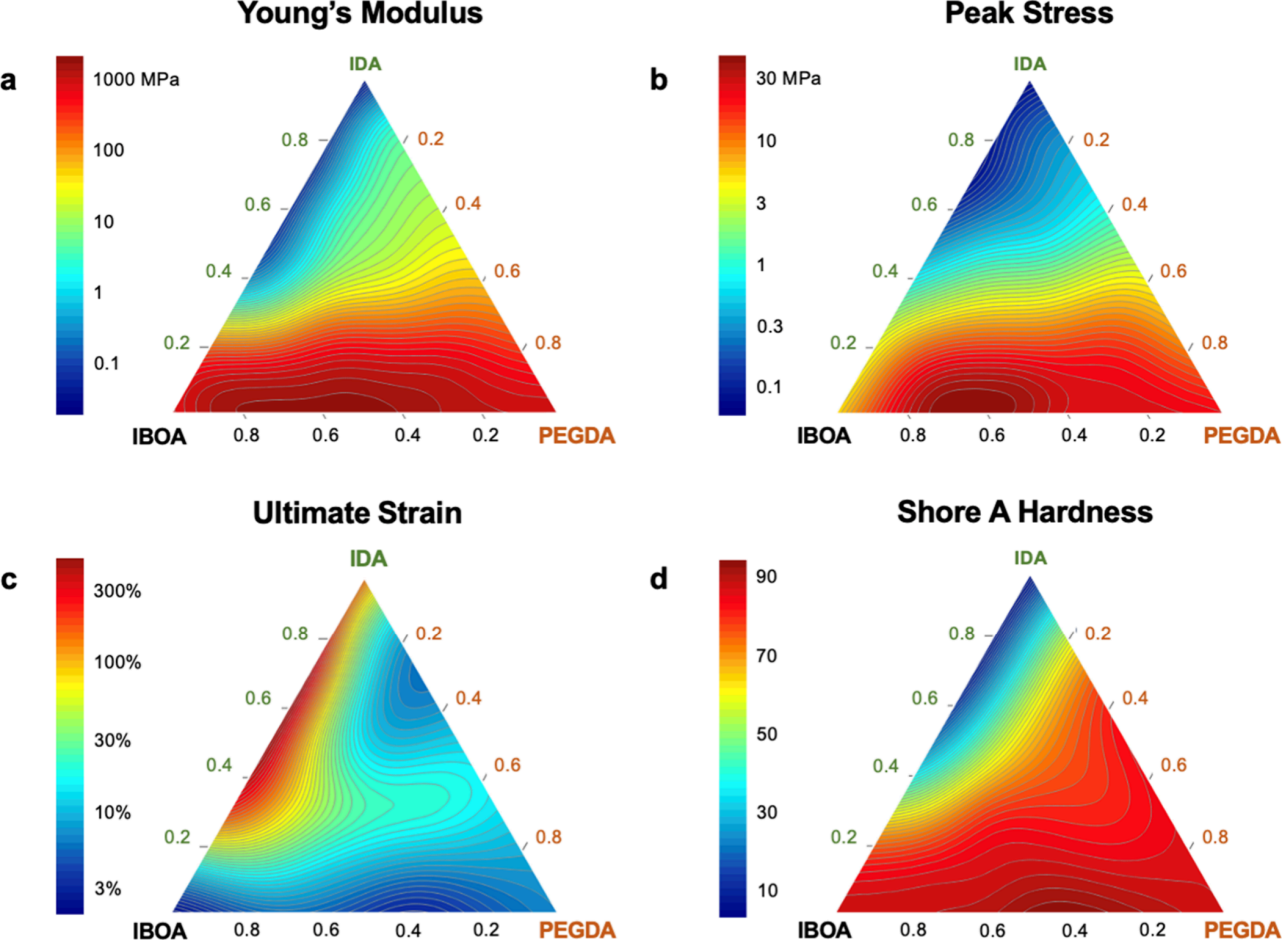
Contour maps of mechanical characteristics predicted by the model after the sixth iteration of the active learning cycle when sufficient data have been accumulated for (a) *E*, (b) σ, (c) ε, and (d) Shore A hardness.

The culminated prediction spaces for each material characteristic after the sixth iteration are presented in Figure 4. Compositions with greater concentrations of IBOA, located in the bottom left corner, exhibit more rigid behavior, with the greatest Young’s moduli, σ, and hardness but inferior ε (Figures 4a–d). This is attributable to the IBOA monomer structure (detailed in Figure 1a), which consists of a bulky, high-molecular-weight functional group, which inhibits chain motion and rotation in addition to its comparably short two carbon contribution to the polymer chain backbone length. Interestingly, as the IDA concentration increases to near 35%, we observe an abrupt decline in *E*, σ, and hardness (Figures 4a,b,d), with an increase in ε (Figure 4c). This indicates that the long 12 carbon IDA monomers begin to dominate the chain structure at this concentration, which allows for greater chain elongation, leading to more elastomeric polymers. This transition becomes less pronounced with the introduction of PEGDA due to its chain cross-linking effects. Cross-linking effects are also observable in ε, which drops even in the relative absence of rigid IBOA (Figure 4c). Moreover, while the hardness contour map (Figure 4d) generally geometrically correlates with the *E* contour map (Figure 4a), the hardness exhibits a consistently acute increase when PEGDA is introduced. This implies that hardness is closely related to *E* at lower concentrations (<20 wt %) of PEGDA. Ultimately, the developed predictive models are superior for extracting insights like the ones discussed versus merely interpolations between experimental results. From Figure S5, we can see that a Cartesian interpolation implemented through the *plotly* library offers a limited view of the characterization space due to more erratic contours, resulting in critical insights potentially being overlooked.

### 3.2 Targeting of Specific Material Characteristic Values (Discovery)

**Figure 5:**
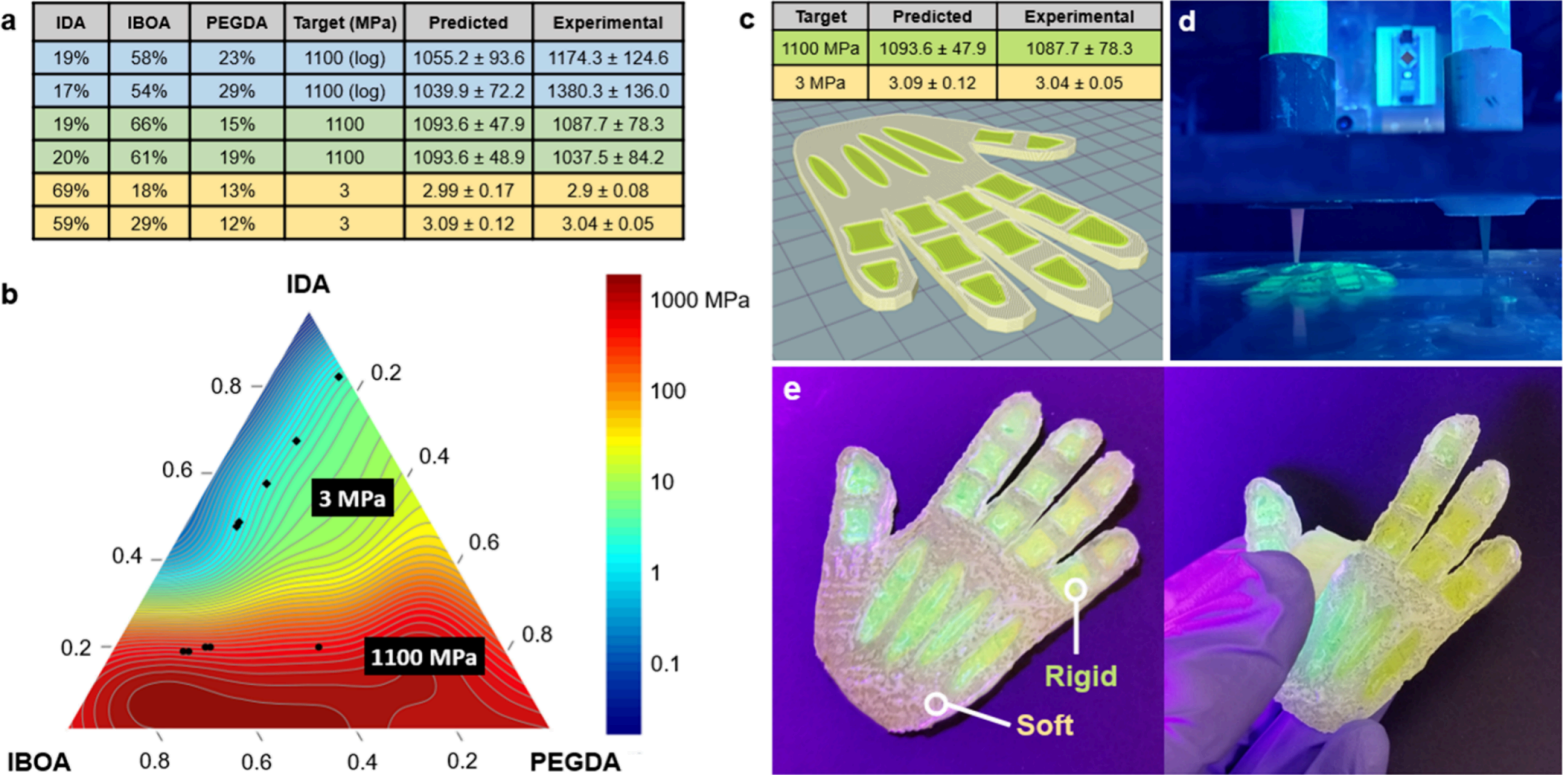
Results of targeted Young’s moduli studies and 3D printing. (a) Selected results of predictions versus experimentally obtained Young’s moduli values. (b) Predicted compositions superimposed on Young’s modulus character space. (c) Mechanical properties of rigid and soft inks and schematic of the structure to be printed after processing in a slicing program. (d) Multimaterial printing of the hand using syringes containing rigid (left) and soft (right) inks. (e) Final printed structure (left) and deformation (right).

We demonstrate the utility of our hierarchical approach to quantitatively suggest compositions meeting target property criteria, detailed in Section 2.4, by applying it to obtain material compositions that exhibit a specified *E* value with up to 10% variability. Simultaneously, we can also account for maximizing or minimizing the ε, ultimate stress, and Shore A hardness. First, we start with a set of candidate compositions. For all compositions, we use the hierarchical approach to predict *E* and a standard GPR model to predict the other three characteristics. Using these predicted bulk characteristics of candidates, we employ exploitation recommendation to obtain five suitable compositions. The predicted material characteristic values versus experimentally obtained values for the recommended compositions are detailed in Figure 5a. For these recommendations, raw predictions and predictions versus experimental values can be found in Tables S1 and S2, respectively. The first material selected has a target of *E* = 1100 ± 100 MPa, with a high σ, high peak strain, and high Shore A hardness, to obtain a composition with high toughness and strength. For comparison, sets of composition predictions for *E* = 1100 ± 100 MPa were generated by using both the logarithmically scaled and hierarchical techniques detailed in Section 2.3. Of the five logarithmically obtained predictions (Table S1, blue), only three had *E* values within the desired range; two predictions were up to 14% away from the *E* = 1100 MPa target (average 8.15%). In contrast, all hierarchically obtained predictions fell within the desired range and averaged within 0.7% of the targeted *E*. Experimental data corroborated the superiority of the hierarchical predictions, which exhibited a 92% reduction in the RMSE versus the logarithmic predictions (8.06 and 102.2 MPa, respectively). Our most accurate *E* prediction was 1093.6 ± 48.9 MPa, which yielded an experimental *E* of 1087.7 ± 78.3 MPa. Additionally, compared to other compositions that had a predicted *E* of 1100 ± 100 MPa, the selected compositions had a 55.7% higher average hypervolume of maximizing Shore A hardness, σ, and ε. The greater hypervolume indicates that our recommended samples have a higher probability of fitting the overall desired characteristics, even when other compositions are within the *E* = 1100 ± 100 MPa range. Despite this, we still find a large discrepancy in the predictions of other characteristics. Notably, the predicted ultimate strain deviates by up to 43.9%. We attribute this to not fine-tuning the other characteristics as we did for Young’s Modulus. This would lead to a decrease in relative accuracy, especially given that the ultimate strains within the dataset span several orders of magnitude. Next, we attempted a target of *E* = 3 ± 0.3 MPa, with maximized σ, ε, and minimized Shore A hardness to obtain a tough, elastomeric material. Three out of five hierarchically obtained compositions were predicted to fall within the desired range and averaged within 1.1% of the targeted *E*. Indeed, Figure 5b indicates the suggested experimental composition predictions closely align with appropriate contours in the model’s *E* prediction space. Moreover, these contours also align with composition values that have a greater predicted hypervolume (Figure S6a, b), specifically 26.8% higher than other compositions with *E* = 3 ± 0.3 MPa, indicating that the Gaussian transformation in Section 2.4 can be used to target certain regions. Interestingly, while the composition predictions have very similar Young’s moduli, they exhibit a diversity of σ and ε values (Figure S6c–e). For example, two compositions in the *E* = 1100 MPa space have nearly identical predicted moduli (*E* = 1093.6 ± 48.9 versus 1093.6 ± 47.9 MPa) yet have a 12% difference in σ (σ = 24.0 ± 2.0 versus 27.1 ± 2.0 MPa) and a 30% difference in ε (ε = 15.8 ± 1.2% versus 12.2 ± 0.9%). Likewise, a similar case can be observed in the *E* = 3 MPa space (*E* = 2.99 ± 0.17 vs 2.99 ± 0.19 MPa), with a 35% difference in σ (σ = 0.31 ± 0.03 vs 0.23 ± 0.01 MPa) and an 88% difference in ε (ε = 18.8 ± 1.2% vs 10.0 ± 0.8%). Intriguingly, the two acquired compositions have radically different material ratios. Despite the far greater percentage of elastomeric IDA in the second composition, the ε is considerably lower than the first (Figure S6d). This is attributable to the observed trend in the prediction space discussed in the previous section wherein ε decreases as IBOA concentration increases when the concentration of PEGDA cross-linker is the same. Conversely, hardness predictions remained highly consistent with *E*, a trend evidenced by the previously discussed similarity of the *E* and hardness prediction space contour map geometries (Figure S6c,f).

Finally, to demonstrate a potential application for this AL methodology, we used experimentally validated *E* = 1100 and 3 MPa compositions to 3D print a multimaterial “hand” (Figure 5c–e). The soft *E* = 3 MPa material (*white*) allows the structure to bend about the finger joints, while the rigid *E* = 1100 MPa material (green) acts as a support, thereby defining distinct joints and maintaining a flat palm area. This demonstrates the viability of our approach as a powerful tool for AM by enabling an informed material selection tailored to user specifications. Material characterization of printed samples will be essential to further demonstrate the validity of predicted materials when applied to resin vat- or extrusion-based photopolymerization 3D printing techniques.

## 4 Conclusions

In this study, we illustrate the efficacy of incorporating active learning alongside a versatile recommendation method, capable of managing noisy training data, for the design of complex, multimonomer polymeric materials tailored for additive manufacturing. Through a systematic and iterative process, we successfully construct a robust dataset and develop advanced machine learning models that can accurately predict the Young’s modulus, peak engineering stress, ultimate strain, and Shore A hardness of 3-component cross-linked polymers. By following a hierarchical approach using a fine-tuned model in regions of interest, we found that we could select compositions within 10% of a desired target value of *E*, while the most optimal candidates fell within 1.1% of the targeted value. Furthermore, the final compositions selected for experimental validation differ by less than 5% from the predicted value. Intriguingly, our predictive model devised resins with an unexpectedly wide range of monomer composition ratios to satisfy the targeted *E* criteria. As a result, predicted resins had noticeable variations in nontargeted characteristics such as peak stress and ultimate strain. The observed combinations of just three monomers demonstrate a wide range of material characteristics, spanning orders of magnitude. Considering the vastness of potential monomer design spaces, future work will be needed to incorporate additional material chemistries. Moreover, this work has potential to engineer materials with superior toughness and/or elastomeric performance; therefore, future work in this direction will involve deeper evaluation of proposed material compositions including hysteresis of cross-linked polymers. We must be careful not to naively apply our approach to these larger design spaces, as our current recommendation assumes that all compositions are realizable. The three monomers studied have relatively high realizabilities, but this may not be the case in expanded design spaces. Therefore, we seek to use a modified approach in which a proxy to composition realizability is predicted and included in the recommendation method. Nevertheless, this study serves as a proof of concept that suggests active learning is a promising method to explore a multimonomer design space, revealing new materials that can satisfy a breadth of applications.

## Supporting Information Available

Additional details regarding the active learning process, dataset, experimental procedures, and additional machine learning model predictions (PDF)

## Notes

We plan to release the data used to train the models at the Ramprasad Group github (https://github.com/Ramprasad-Group).
