# Supplementary material for: Machine-Guided Discovery of Acrylate Photopolymer Compositions
Source: ACS Appl Mater Interfaces. 2024 Mar 27;16(14):17992–8000. doi: 10.1021/acsami.4c00759 (PMC11009904; doi:10.1021/acsami.4c00759)
Supplement: Supplementary file 1 — am4c00759_si_001.pdf [file am4c00759_si_001.pdf]

Supporting Information

Machine-Guided Discovery of Acrylate Photopolymer Compositions

Ayush Jain[1,2$], Connor D. Armstrong[3,4$], V. Roshan Joseph[5], Rampi Ramprasad[1]*, H. Jerry Qi[3,4]*

[1]School of Material Science and Engineering, Georgia Institute of Technology, Atlanta, GA 30332, USA

[2]College of Computing, Georgia Institute of Technology, Atlanta, GA 30332, USA

[3]School of Mechanical Engineering, Georgia Institute of Technology, Atlanta, GA 30332, USA

[4]Renewable Bioproducts Institute, Georgia Institute of Technology, Atlanta, GA, 30332, USA

[5]School of Industrial and Systems Engineering, Georgia Institute of Technology, Atlanta, GA, 30332, USA

[$] These two authors made equal contributions

*Corresponding authors: RR: rampi.ramprasad@mse.gatech.edu; HJQ, qih@me.gatech.edu;

Keyword: Photopolymers, Active Learning, Gaussian Process Modeling, Material Characterization, Predictive Algorithms;

Supplementary Materials:

Figures S1, S2, S3, S4, S5

Table S1, S2

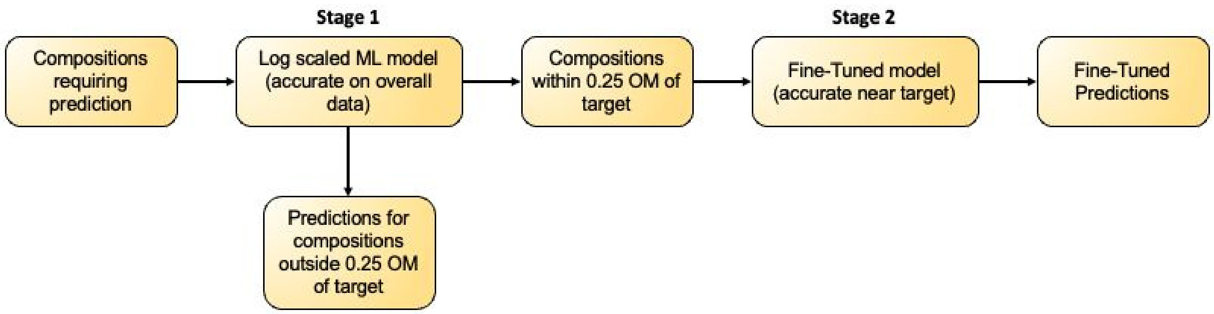

Figure S1. Process flow diagram for the hierarchical targeted prediction approach.

Round	Goal	Recommendation Method
0	Initial Data	N/A
1-5	Model Building	NEHVI
6	Model Building	Exploration
7	Model Testing	Random
8	Model Testing	Hand-Selected
9	Targeted Young's Modulus (Trial)	NEHVI
10 A	Targeted Young's Modulus 1100 +/- 100 MPa	Exploitation (log)
10 B	Targeted Young's Modulus 1100 +/- 100 MPa	Exploitation (hierarchical)
11	Targeted Young's Modulus 3 MPa +/- 0.3 MPa	Exploitation (hierarchical)

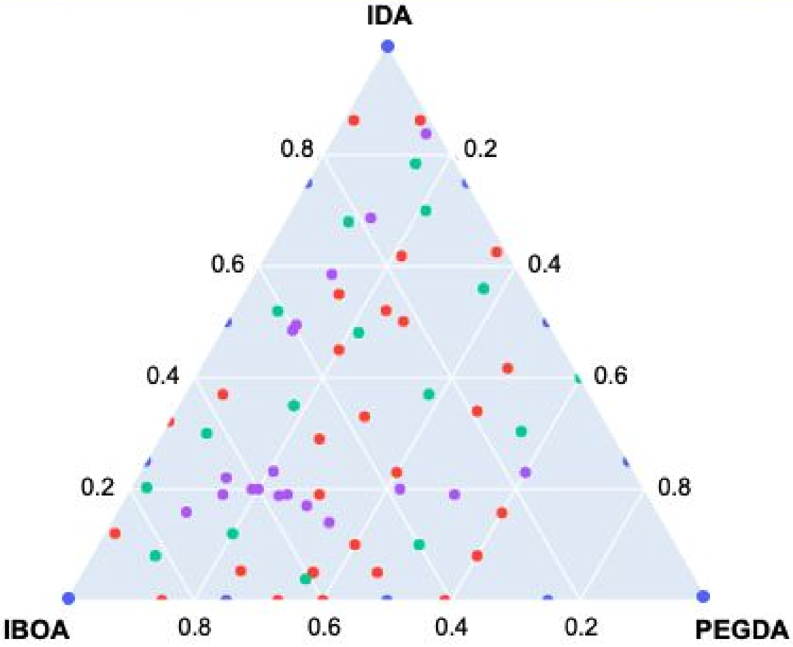

Figure S2. Overview of all experiments undertaken.

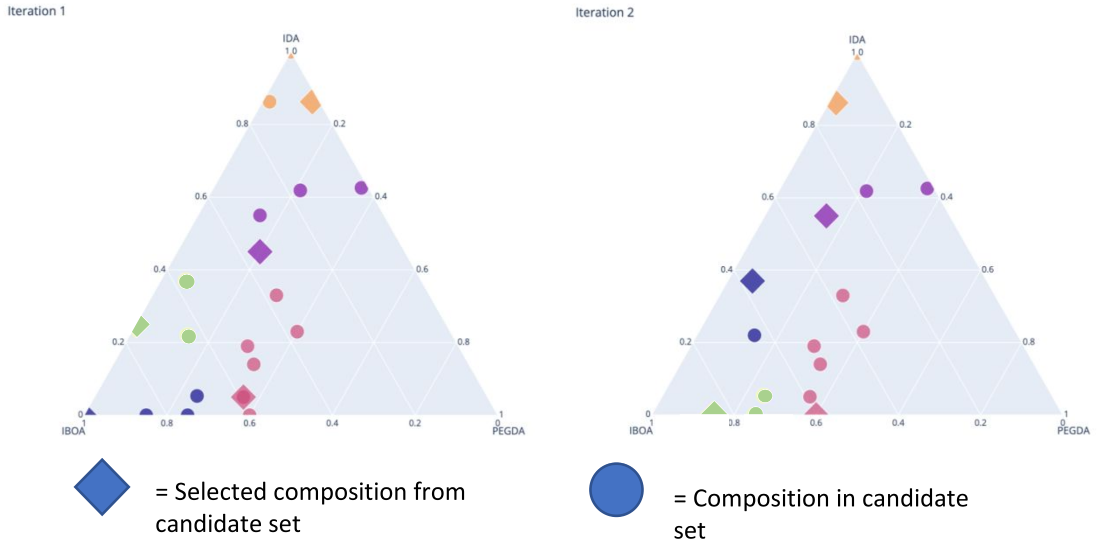

Figure S3: A depiction of the Random Grid Search across an iteration of AL, with each composition in the candidate space represented by circles and diamonds.

The core reasoning for using Random Grid Search is to simulate the possibilities of a person visually handpicking samples which are spaced apart from each other at every iteration. As explained in **Section 3.1** of the main text, we do not have the ability to create and test every possible sample, so we use a candidate set of compositions to pick from for this grid search.

We now describe the strategy for "random grid search." Note that a "point" in this context is defined as a composition ratio denoted as (%IDA, %IBOA, %PEGDA), and the composition space as being in Euclidean space.

Consider a scenario where we have a candidate set X and want to get 5 points in one iteration of active learning. We initiate a K-means clustering algorithm to cluster all points in X into 5 clusters, with the intention of having a small Euclidean distance between points in the cluster. In **Figure S3**, the points in each cluster (x_p) are signified by different colors, and one can note that points with similar composition values are grouped together. In every cluster, we randomly select a point. We then end up with 5 points that are random but are theoretically very different from each other, mimicking an uninformed random selection.

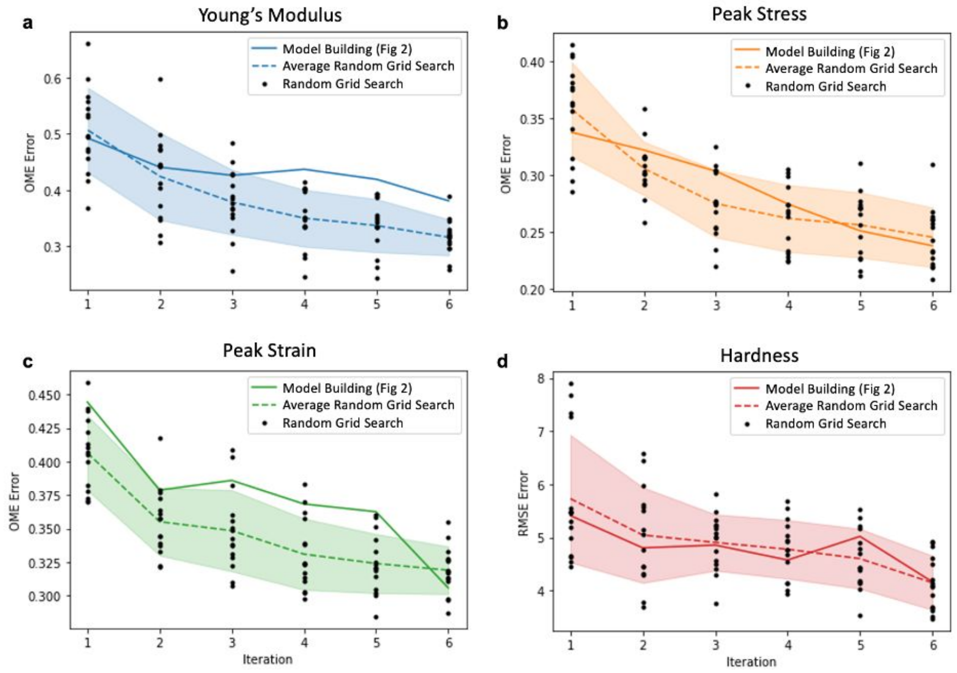

Figure S4. Accuracy evolution of predictive models over the six training iterations. OME used for **(a)** Young's modulus, **(b)** peak stress, and **(c)** ultimate strain. **(d)** RMSE evolution over six training iterations for hardness. The block dots are representative of errors from all Random Grid Search trials. The shaded area is standard deviation of these trials.

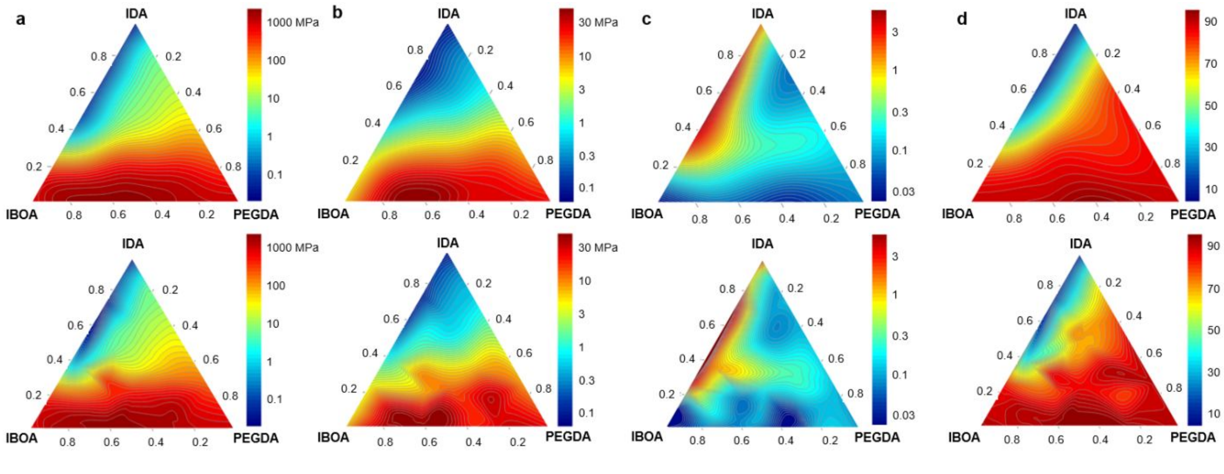

Figure S5. Contour maps of mechanical characteristics at round 6 generated via predictive model (*top*) versus experimental interpolation (*bottom*) for **(a)** Young's Modulus, **(b)** peak stress, **(c)** peak strain, and **(d)** Shore A hardness.

IDA	IBOA	PEGDA	E Target	Young's Modulus (MPa)		Peak Stress (MPa)		Ultimate Strain (mm/mm)		Shore A Hardness	
				Predicted	±	Predicted	±	Predicted	±	Predicted	±
19	58	23	1100 ± 100 (log)	1055.173646	93.62765417	25.59500363	1.964030062	0.170678028	0.014436636	83.66500316	1.123491779
17	54	29		1039.950809	72.16978898	26.86213399	1.469521206	0.15654292	0.01298704	85.41588376	0.965360653
19	56	25		958.7469997	82.27391742	24.12247714	1.728164132	0.179963421	0.015201363	84.13367554	1.059684771
17	56	27		1145.401632	87.99620399	28.40694113	1.771545087	0.150289522	0.012593824	85.08480748	1.011261966
17	53	30		943.0609213	58.4369549	25.43214187	1.207347044	0.162457305	0.013346599	85.72412968	0.940339692
20	61	19	1100 ± 100	1093.605474	48.88271403	23.95635189	2.021968661	0.157654176	0.011991524	81.04351842	1.267570691
20	60	20		1108.91587	48.38744903	23.75320752	2.011577304	0.158758774	0.012232142	81.66222024	1.223477035
19	66	15		1093.594001	47.94243443	27.14545468	2.027556033	0.121659702	0.009108319	81.31309009	1.273737224
19	46	17		1111.945162	46.57639302	28.32445961	2.191523625	0.128378291	0.009699766	81.71978161	1.273372763
20	38	42		1104.572408	42.38354306	17.73145326	1.180099351	0.1121544	0.007871503	85.72813492	1.297953598
49	40	11	3 ± 0.3	1.872961193	0.127913613	0.52717462	0.041928684	0.313886258	0.018924648	47.23433192	1.272245508
59	29	12		2.993543688	0.168402075	0.305719594	0.02700759	0.187382117	0.012445518	44.93643335	1.203731716
69	18	13		3.088239537	0.11719397	0.233577545	0.023506718	0.135465429	0.010029208	45.32816662	1.397616764
48	41	11		1.769896052	0.124868066	0.56805555	0.045771826	0.33401463	0.020142418	47.92606126	1.282913135
84	2	14		2.99185457	0.195654775	0.233619228	0.005222414	0.099894901	0.008438204	45.56571631	1.658145295

Table S1. All predictions of recommended compositions for targeted Young's moduli studies.

IDA	IBOA	PEGDA	E Target	Young's Modulus (MPa)		Peak Stress (MPa)		Ultimate Strain (mm/mm)		Shore A Hardness	
				Predicted	Experimental	Predicted	Experimental	Predicted	Experimental	Predicted	Experimental
19	58	23	1100 ± 100 (log)	1055.173646	1174.305131	25.59500363	27.97142857	0.170678028	0.070857143	83.66500316	82
17	54	29		1039.950809	1380.320261	26.86213399	29.5	0.15654292	0.114875	85.41588376	87.41666667
20	61	19	1100 ± 100	1093.605474	1037.472333	23.95635189	21.03333333	0.157654176	0.088666667	81.04351842	87.25
19	66	15		1093.594001	1087.692715	27.14545468	21.25714286	0.121659702	0.075857143	81.31309009	85
59	29	12	3 ± 0.3	2.993543688	2.8588	0.305719594	0.28	0.187382117	0.1664	44.93643335	46.6
69	18	13		3.088239537	3.0385	0.233577545	0.325	0.135465429	0.14015	45.32816662	49.6

Table S2. Prediction versus experimental results of recommended compositions for targeted Young's moduli studies.

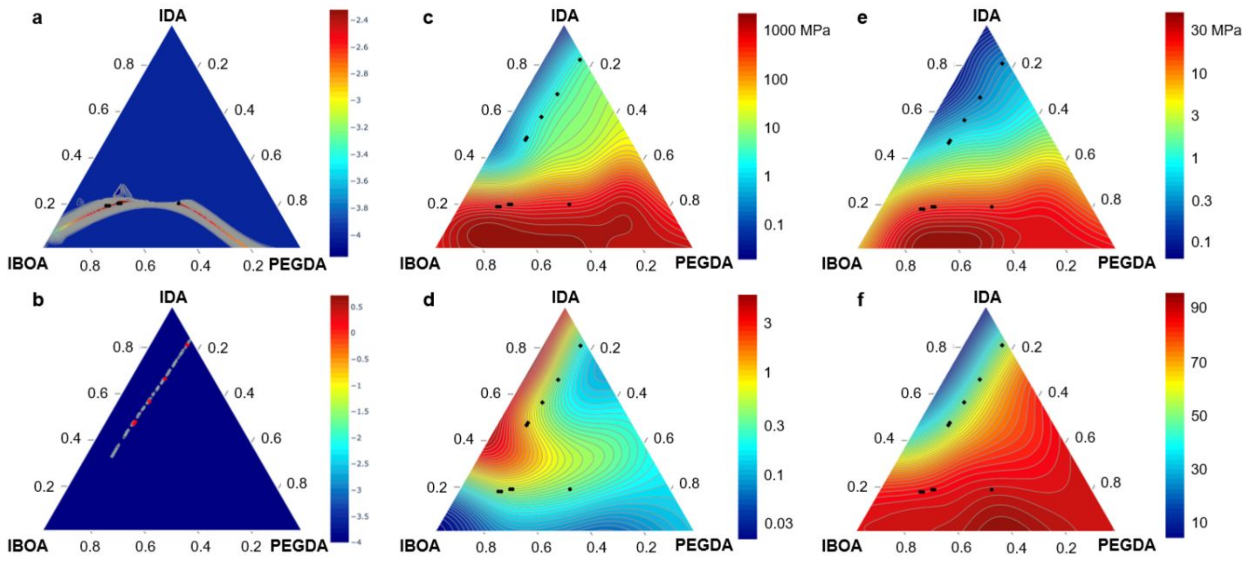

Figure S6. Results of targeted Young's moduli studies. Hypervolume heatmaps of targeted Young's modulus predictions for **(a)** 1100 MPa (*black)* and **(b)** 3MPa (*red*). Predicted compositions superimposed on **(c)** Young's modulus, **(c)** peak stress, **(d)** peak strain, and **(d)** hardness character spaces. Note that in (a) and (b), changes in hypervolume occur drastically when compositions have a predicted E that is farther from the target. Visually this appears as dense contour lines forming a "grey" area.
